# Electrocardiogram P-Wave Dispersion and New-Onset Atrial Fibrillation: A Nested Case–Control Study

**DOI:** 10.31083/RCM45617

**Published:** 2026-02-11

**Authors:** Chunlei Wu, Yunxia Ma, Pan Yang, Ying Li, Min Xu

**Affiliations:** ^1^Department of Electrocardiography and Cardiology, The Third Affiliated Hospital of Soochow University, 213000 Changzhou, Jiangsu, China; ^2^Department of Echocardiography and Cardiology, The Third Affiliated Hospital of Soochow University, 213000 Changzhou, Jiangsu, China

**Keywords:** electrocardiogram, P-wave dispersion, atrial fibrillation, risk

## Abstract

**Background:**

This study aimed to investigate the association between electrocardiogram (ECG) P-wave dispersion (Pd) in sinus rhythm and the risk of new-onset atrial fibrillation (NAF) within one year, to identify high-risk individuals earlier and improve clinical outcomes.

**Methods:**

(1) This retrospective nested case–control study included patients diagnosed with NAF at Changzhou First People's Hospital between July 2022 and June 2023. Cases were defined as individuals without a previous atrial fibrillation (AF) diagnosis who developed NAF regardless of symptom status. Controls were matched 1:3 by age and sex from individuals with sinus rhythm during the same period. (2) Using the date of the NAF diagnosis as the index date, then ECGs, echocardiographic data, laboratory tests, and basic clinical characteristics in the sinus rhythm were collected via the electronic medical record system for all subjects within one year post-index date. (3) Differences in ECG parameters, echocardiographic parameters, blood biological indicators, and basic clinical characteristics in the sinus rhythm were compared between the two groups. (4) Conditional logistic regression models were used to evaluate the association between relevant ECG indicators and NAF, with curve fitting performed using generalized additive models (GAMs).

**Results:**

(1) A total of 824 participants were enrolled, including 206 NAF cases and 618 matched controls. (2) A comparison between groups identified significantly higher diastolic blood pressure, glycated hemoglobin A1c, serum creatinine, P-wave duration, Pd, and left atrial diameter in the NAF group than the control group; meanwhile, uric acid, total cholesterol, high-density lipoprotein, and low-density lipoprotein were significantly lower (all *p* < 0.05). (3) In fully adjusted conditional logistic regression models, increased Pd was independently associated with a higher risk of NAF within one year (odds ratio (OR): 1.149; 95% confidence interval (CI): 1.099–1.202; *p* < 0.001). Curve fitting demonstrated a positive correlation between Pd and the risk of NAF.

**Conclusions:**

ECG Pd in the sinus rhythm was independently and positively associated with the risk of NAF within one year.

## 1. Introduction

Atrial fibrillation (AF) is the most common sustained cardiac arrhythmia in clinical practice, with rising global prevalence that increases markedly with age [1]. As a progressive disease, AF increases the risk of ischemic stroke by five-fold [2], and is associated with an elevated risk of heart failure, cognitive impairment, myocardial infarction, and sudden cardiac death [3], posing serious threats to patients’ health and quality of life while imposing substantial burdens on socioeconomic systems and healthcare services. It is reported that about one-third of AF patients are asymptomatic, especially in the early stages of recognition, while the clinical outcomes of asymptomatic AF patients show no significant difference compared to symptomatic patients [4]. Therefore, identifying patients at risk for AF is crucial for its early prevention, diagnosis, and treatment.

Several risk schemes have been proposed to predict new-onset atrial fibrillation (NAF), including the Cohorts for Heart and Aging Research in Genomic Epidemiology-Atrial Fibrillation (CHARGE-AF) score [5], the Mayo AF score [6], and the HARMS2-AF score, using hypertension, age, raised body mass index (BMI), male sex, sleep apnea, smoking, and alcohol for assessment [7]. However, these scoring schemes were derived in European and American populations, and may not be fully applicable to Asian patients. The onset and maintenance of AF depend on electrical and structural remodeling of the atria [8]. Previous studies have shown that electrocardiographic P-wave indices can reflect underlying atrial remodeling [9]. The P-wave dispersion (Pd) represents the degree of variation in P-wave duration (PWD) across the 12 surface leads of the electrocardiogram (ECG) in sinus rhythm, reflecting the presence of spatially heterogeneous electrical activity within the atria. Multiple studies have demonstrated that increased Pd is independently associated with the occurrence of paroxysmal atrial fibrillation (PAF) [10, 11]. However, there is insufficient evidence to support an association between Pd and NAF in population-based observational studies. In this study, we conducted a retrospective analysis of the most recent sinus rhythm Pd within the preceding year in patients with NAF, using a simple, cost-effective, noninvasive, and widely accessible 12-lead surface ECG. The aim was to investigate the association between Pd and NAF within one year.

## 2. Methods

### 2.1 Study Design and Population

**Fig. 1.**
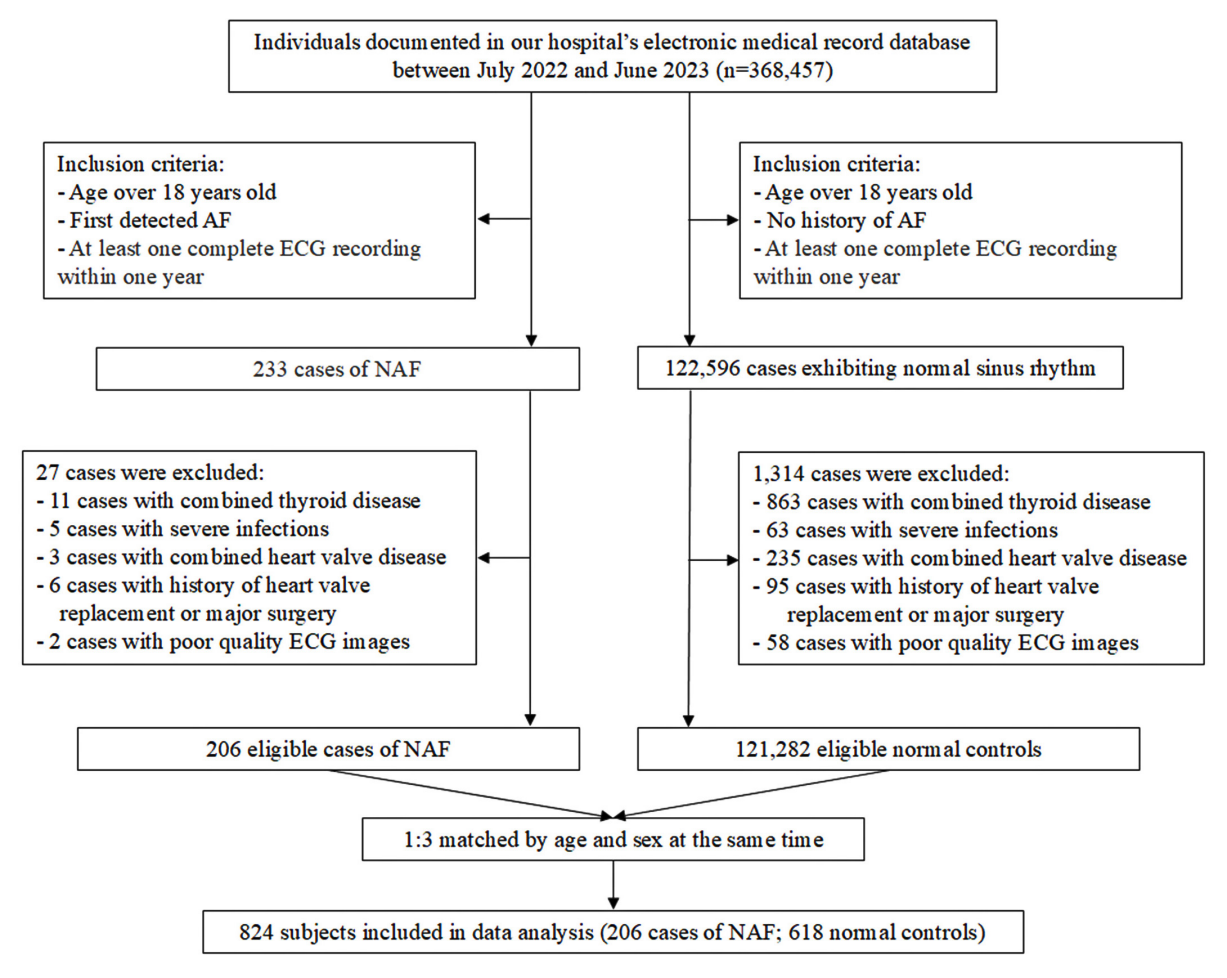
**Flowchart of the study**. AF, atrial fibrillation; ECG, electrocardiogram; NAF, new-onset atrial fibrillation.

This was a retrospective nested case-control study utilizing data from the electronic medical record system at the Third Affiliated Hospital of Soochow University, which included 368,457 subjects between July 2022 and June 2023. Patients with NAF were assigned to the case group. The inclusion criteria for the NAF group were as follows: (1) Age over 18 years old; (2) AF not diagnosed before, irrespective of its duration or the presence/severity of AF-related symptoms. Confirmation of AF via 12-lead ECG (≥10 seconds) or single-lead ECG (≥30 seconds) showing absent P-waves and irregular RR intervals (with preserved atrioventricular conduction) [12]; (3) At least one complete ECG recording within the preceding year. Controls were selected from the same database at a 1:3 ratio matched by age and sex, consisting of subjects with sinus rhythm during the same period. The inclusion criteria for the control group were as follows: (1) Age over 18 years old; (2) No history of AF; (3) At least one complete ECG recording within the preceding year. The common exclusion criteria for both groups were as follows: (1) Thyroid dysfunction; (2) Severe infection; (3) Valvular heart disease; (4) Prior valve replacement or major cardiac surgery; (5) Poor-quality ECG tracings. The study adhered to the principles of the Declaration of Helsinki and was approved by the Scientific Ethics Committee of the Third Affiliated Hospital of Soochow University (Approval No: [2022] Tech-018). Informed consent was waived due to the retrospective nature of the study. The study flowchart is shown in Fig. 1.

### 2.2 Collection of Clinical Data 

In this study, the diagnosis date of NAF was defined as the index date. Through the electronic medical record system, we collected ECG indicators in sinus rhythm status within one year prior to the index date for the study subjects, concurrently gathering clinical data. The variables included: socio-demographic data, including age and sex; lifestyle factors and physiological parameters, such as smoking, alcohol consumption, BMI, and blood pressure; comorbidities, such as hypertension, diabetes mellitus (DM), coronary heart disease (CHD), and stroke; laboratory parameters, such as fasting glucose, lipid profile, serum creatinine (SCR), and uric acid (UA); use of cardiovascular medications such as angiotensin-converting enzyme inhibitors/angiotensin receptor blockers (ACEIs/ARBs), beta-blockers, calcium channel blockers (CCBs), statins, and antiplatelet agents; as well as echocardiographic parameters including left atrial diameter (LAD), interventricular septal thickness (IVS), left ventricular end-diastolic diameter (LVEDD), and left ventricular ejection fraction (LVEF). Echocardiographic measurements were performed using the Philips EPIQ 7c (Release 5.2.2, Philips Healthcare Royal Philips Electronics, Amsterdam, the Netherlands) colour Doppler echocardiographer..

### 2.3 Acquisition of ECG Parameters

Standard 12-lead ECG in sinus rhythm were collected from the study subjects within the last preceding year through the electronic medical record system. Recordings were acquired using a FX-8322 12-channel electrocardiograph (V03-03, Beijing Futian Electronic Medical Instruments Co., Ltd., Beijing, China) at 25 mm/s paper speed and 10 mm/mV calibration. Participants were in supine position and at rest during recording. Automated ECG parameters included heart rate (HR), PR interval (PR), PWD, and P-wave voltage in lead I (PVL I). Subsequently, two independent cardiac electrophysiologists processed the 12-lead synchronous ECGs through digitization involving 300 DPI scanning and 10-fold magnification. They used a blind method to measure the indices Pd and P-wave peak time in lead II (PWPT II). Novel ECG parameters, including Pd and PWPT II, were measured based on methods in the previous study [13]. P-wave onset and offset were identified as the intersection points between the P-wave contour and the isoelectric baseline (upper tangent method). The duration between these two points constituted the PWD. Each measurement was repeated three times per lead and averaged. At least eight leads were analyzed per ECG. The Pd was the difference between the maximum PWD and the minimum PWD. The PWPT II was the time from the P-wave onset to the peak of the P-wave in Lead II, averaged after measuring three consecutive cardiac cycles.

### 2.4 Statistical Analysis

EmpowerStats software (https://www.empowerstats.net/cn/, version 3.3.0, X&Y Solutions, Inc, Boston, MA, USA) and R (version 3.4.3, R Foundation for Statistical Computing, Vienna, Austria) were used for the statistical analysis. Continuous variables were tested for normality using the Kolmogorov-Smirnov test (K-S test). Data were considered normally distributed when the *p*-value was ≥0.05 (R1). Normally distributed data were expressed as mean ± standard deviation (SD), while non-normally distributed data were presented as median with interquartile range (IQR). Categorical variables were presented as frequencies (%). Group comparisons were made using chi-square tests (categorical variables), Kruskal-Wallis test (continuous variables), and Fisher exact test when expected cell counts were <10. Collinearity was examined using the variance inflation factor (VIF) stepwise selection method (with VIF <10).

To investigate the association between ECG Pd in sinus rhythm and NAF within one year, three models were employed for association estimation: Model 1 (unadjusted); Model 2 (preliminary adjustment) adjusted for age, sex, BMI, diastolic blood pressure (DBP), and HR; and Model 3 (full adjustment) further adjusted for glycated hemoglobin A1c (HbA1c), SCR, UA, total cholesterol (TC), high-density lipoprotein (HDL), low-density lipoprotein (LDL), and LAD. In conditional logistic regression analyses, covariates were retained in the final model if they changed the regression coefficient of Pd by >10% or showed a significant association with NAF (*p* < 0.01). Generalized additive models (GAMs) were used to explore potential nonlinear associations between Pd and NAF risk, adjusting for all aforementioned covariates. A *p* < 0.05 was considered statistically significant.

## 3. Results

### 3.1 Characteristics of the Study Subjects

From July 2022 to June 2023, this study consecutively enrolled 233 patients with NAF. After excluding 27 due to comorbidities or poor ECG quality, 206 cases were included. Among these, 119 (57.767%) were male, with a mean age of 71.398 ± 10.546 years. Concurrently, 618 controls with sinus rhythm were selected from our hospital’s electronic medical record database, including 358 males (57.929%) with a mean age of 71.146 ± 7.264 years. Compared to controls, the NAF group exhibited significantly higher values in DBP, HbA1c, SCR, PWD, Pd, and LAD. Conversely, levels of UA, TC, HDL, and LDL were significantly lower in the NAF group (all *p* < 0.05, Table 1).

**Table 1.** **Characteristics comparison between NAF and sinus rhythm groups**.

Characteristics		NAF	Controls	*p*-value
		(n = 206)	(n = 618)	
Age (years)		71.398 ± 10.546	71.146 ± 7.264	0.219
BMI (kg/m^2^)		23.726 ± 3.767	23.992 ± 2.719	0.107
Male, n (%)		119 (57.767)	358 (57.929)	0.968
HBP, n (%)		82 (39.806)	217 (35.113)	0.242
DM, n (%)		27 (13.107)	70 (11.330)	0.624
CHD, n (%)		24 (11.650)	55 (8.899)	0.274
Stroke, n (%)		10 (4.854)	20 (3.236)	0.286
ACHO, n (%)		27 (13.107)	88 (14.239)	0.685
Drink, n (%)		38 (18.447)	85 (13.754)	0.114
SBP (mmHg)		135.922 ± 21.304	135.427 ± 17.546	0.709
DBP (mmHg)		76.607 ± 12.249	73.375 ± 9.649	<0.001
GLU (mmol/L)		6.247 ± 2.367	8.783 ± 3.141	0.179
HbA1c (%)		6.805 ± 3.588	6.134 ± 0.821	0.008
SCR (µmol/L)		92.846 ± 87.902	73.044 ± 24.309	0.035
UA (µmol/L)		332.347 ± 112.593	349.490 ± 85.721	0.022
TC (mmol/L)		4.296 ± 1.296	5.148 ± 1.123	<0.001
TG (mmol/L)		1.484 ± 1.302	1.619 ± 1.033	0.172
HDL (mmol/L)		1.162 ± 0.343	1.388 ± 0.368	<0.001
LDL (mmol/L)		2.483 ± 0.984	3.010 ± 0.898	<0.001
HR (bpm)		71.597 ± 14.067	73.515 ± 12.279	0.062
PVL I (mV)		0.040 (0.030–0.060)	0.040 (0.030–0.060)	0.098
PWPT II (ms)		58.752 ± 10.351	57.793 ± 7.706	0.151
PWD (ms)		117.519 ± 14.892	115.531 ± 9.845	0.033
Pd (ms)		61.301 ± 23.084	28.180 ± 9.621	<0.001
PR (ms)		170.873 ± 34.895	164.540 ± 23.977	0.078
LVEF (%)		62.597 ± 6.200	63.191 ± 3.204	0.582
LAD (mm)		39.805 ± 5.639	35.040 ± 3.335	<0.001
IVS (mm)		9.816 ± 1.308	9.609 ± 0.976	0.687
LVEDD (mm)		49.408 ± 5.025	48.372 ± 3.461	0.347
Medications, n (%)				
	Antiplatelets	15 (7.282)	49 (7.929)	0.881
	ACEIs/ARBs	60 (29.126)	165 (26.699)	0.528
	Beta-blocker	45 (21.845)	100 (16.181)	0.073
	CCBs	43 (20.874)	96 (15.534)	0.086
	Statin	50 (24.272)	175 (28.317)	0.279
	Oral hypoglycemic agents	10 (5.834)	30 (4.854)	0.580
	Insulin	15 (7.282)	55 (8.900)	0.564

The results are expressed as mean ± SD/n (%). BMI, body mass index; HBP, high blood pressure; DM, diabetes mellitus; CHD, coronary heart disease; ACHO, alcohol; SBP, systolic blood pressure; DBP, diastolic blood pressure; GLU, glucose; HbA1c, glycated hemoglobin A1c; SCR, serum creatinine; UA, uric acid; TC, total cholesterol; TG, triglycerides; HDL, high-density lipoprotein; LDL, low-density lipoprotein; bpm, beats per minute; HR, heart rate; PVL Ⅰ, P-wave voltage in lead I; PWPT Ⅱ, P-wave peak time in lead II; PWD, P-wave duration; Pd, P-wave dispersion; PR, PR interval; LVEF, left ventricular ejection fraction; LAD, left atrial diameter; IVS, interventricular septum; LVEDD, left ventricular end-diastolic diameter; ACEIs/ARBs, angiotensin-converting enzyme inhibitors or angiotensin receptor blockers; CCBs, calcium channel blockers.

### 3.2 Conditional Logistic Regression Analysis 

To further investigate, this study adjusted for potential confounding factors and performed multivariate regression analyses. The unadjusted model was equivalent to univariate analysis. The preliminary adjusted model (Adjustment I) included age, sex, BMI, DBP, and HR, while the fully adjusted model (Adjustment II) further incorporated HbA1c, SCR, UA, TC, HDL, LDL, and LAD. The results demonstrated that the continuous variable Pd was significantly associated with the risk of NAF in the unadjusted, Adjustment I, and Adjustment II regression models (ORs were 1.126, 1.127, and 1.149, respectively; all *p* < 0.001) (Table 2).

**Table 2.** **Conditional logistic regression analysis of ECG indicators for predicting NAF**.

Exposure	Non-adjusted	Adjust I	Adjust II
HR	0.988 (0.974, 1.002) 0.093	0.987 (0.973, 1.001) 0.078	0.978 (0.951, 1.005) 0.106
PWD	1.016 (0.998, 1.033) 0.076	1.017 (0.999, 1.035) 0.064	1.038 (1.004, 1.073) 0.029
PWPT II	1.014 (0.992, 1.036) 0.221	1.014 (0.992, 1.036) 0.217	1.041 (0.987, 1.099) 0.137
Pd	1.126 (1.107, 1.145) <0.001	1.127 (1.109, 1.146) <0.001	1.149 (1.099, 1.202) <0.001
PR	1.008 (1.002, 1.014) 0.006	1.008 (1.003, 1.014) 0.004	1.007 (0.987, 1.027) 0.497

The results are expressed as β (95% CI) *p*-value/OR (95% CI) *p-*value. HR, heart rate; PWD, P-wave duration; PWPT Ⅱ, P-wave peak time in lead II; Pd, P-wave dispersion; PR, PR interval. The unadjusted model was equivalent to univariate analysis; the preliminary adjusted model included age, sex, BMI, DBP, and HR; the fully adjusted model included age, sex, BMI, DBP, HR, HbA1c, SCR, UA, TC, HDL, LDL, and LAD.

### 3.3 Curve Fitting

**Fig. 2.**
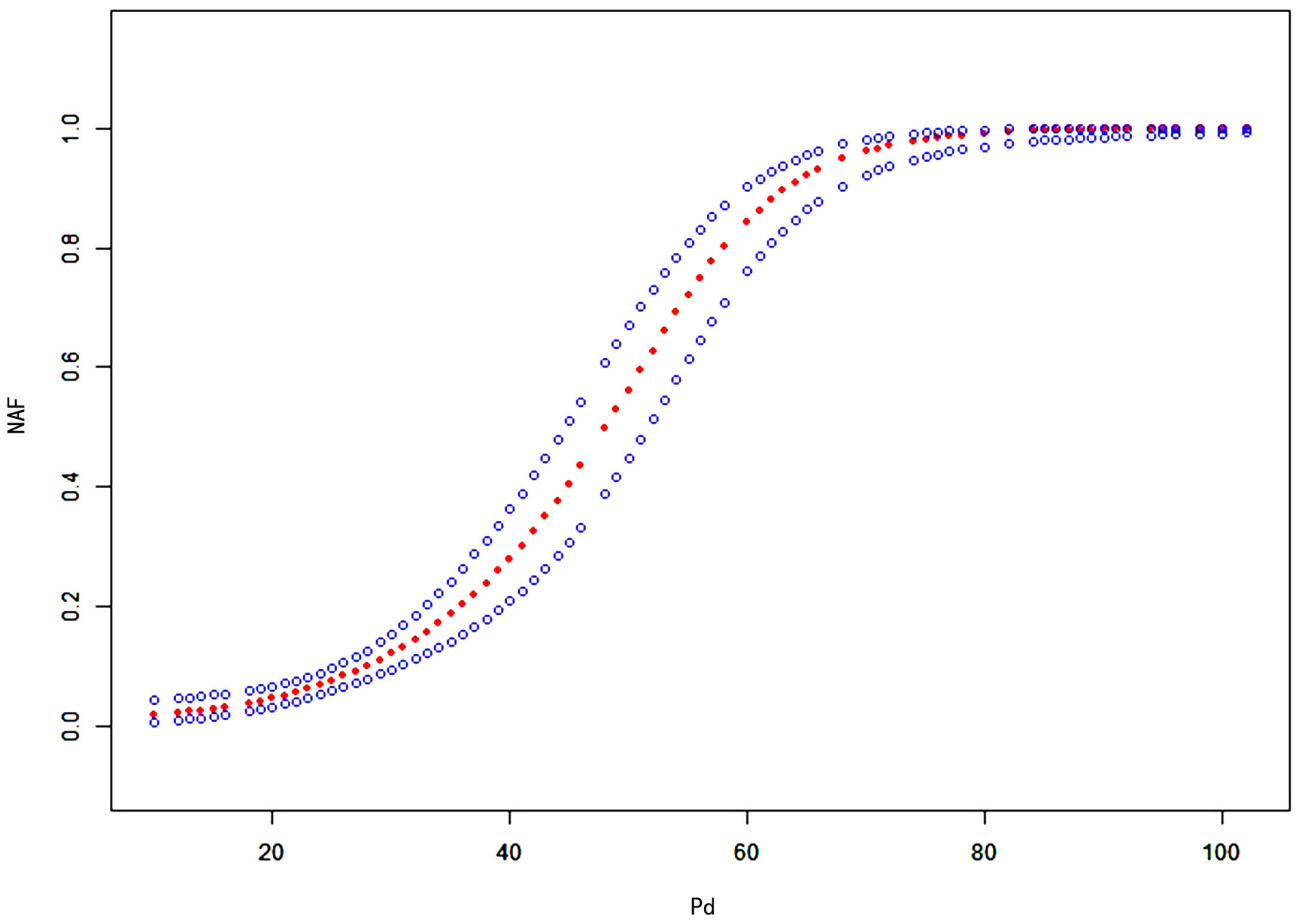
**Curve fitting analysis of the relationship between Pd in sinus rhythm and risk of NAF within one year**. The x-axis represents Pd (continuous variable), and the y-axis represents the risk of NAF (0 = absent, 1 = present). The red dashed line shows the fitted curve between Pd and the risk of NAF, and the blue dashed lines represent the 95% confidence interval. Pd, P-wave dispersion; NAF, new-onset atrial fibrillation.

GAMs were used to test the association between Pd in sinus rhythm and the risk of NAF within one year. After adjusting for covariates (age, sex, BMI, DBP, HbA1c, SCR, UA, TC, HDL, LDL, HR, and LAD), the results showed that the risk of NAF progressively increased with higher Pd values, revealing a significant nonlinear positive correlation (degrees of freedom: 1.000; *p* < 0.001, Fig. 2).

## 4. Discussion

This study revealed that patients who developed NAF exhibited significantly higher Pd values on sinus rhythm ECGs during the preceding year compared to controls. Following comprehensive adjustment for potential confounders, Pd maintained a significant association with NAF within one year. Moreover, the GAM analysis demonstrated a nonlinear positive correlation between Pd and NAF risk.

AF represents a growing global health challenge with complex pathophysiology. Both non-modifiable factors (such as advanced age, genetic predisposition, and sex differences) and modifiable factors (such as HBP, DM, smoking, obesity, physical inactivity, and obstructive sleep apnea) contribute to increased AF susceptibility. Inflammation, oxidative stress, autonomic nervous system dysregulation, and activation of the renin-angiotensin-aldosterone system (RAAS) can induce both electrical and structural remodeling of the atria, thereby promoting both the initiation and perpetuation of AF [8]. Early risk prediction, screening, diagnosis, and individualized management are important for patients to reduce complications and improve quality of life. Therefore, population-based studies to explore the predictive value of noninvasive diagnostic techniques for NAF have long been of interest. The CHARGE-AF scheme [5] was an AF prediction model developed using individual-level data from 3 large cohorts in the United States (the Atherosclerosis Risk in Communities [ARIC] study, the Cardiovascular Health Study [CHS], and the Framingham Heart Study [FHS]), and validated in two European cohorts. The predictive performance of the simple and enhanced scores was comparable (AUC = 0.765 vs 0.767). The Mayo AF score [6] was an AF model developed based on seven recognized clinical risk factors. For patients with AF risk scores of 1, 2, 3, 4, 5 and above, the OR values for subsequent diagnosis of AF were 3.05, 12.9, 22.8, 34.0, 48.0, respectively, and AUC = 0.812. The HARMS2-AF score [7] was a novel lifestyle risk scoring scheme developed and validated using data from the UK Biobank (UKB) and the FHS. Over a 5-year follow-up period, the AUC was 0.782 in the UKB cohort and 0.757 in the FHS validation cohort. The above scoring schemes demonstrate good predictive efficacy, but their applicability to Asian populations remains to be validated due to differences in ethnicity and lifestyle. In addition to simple risk scoring models constructed from cardiovascular risk factors associated with AF, machine learning prediction models driven by artificial intelligence have also been rapidly advancing in recent years. Lubitz *et al*. [14] demonstrated that wearable devices might facilitate identifying individuals with undiagnosed AF. Raghunath *et al*. [15] used a deep network learning approach based on artificial intelligence techniques to predict NAF from resting 12-lead ECG. However, the high demands on signals, data, and decision models may limit their widespread clinical application.

As is well known, the simpler an AF risk assessment tool is, the easier it becomes to identify high-risk patients early on, thereby facilitating further monitoring and diagnosis of AF. This study employed a conventional 12-lead ECG, where the P-wave represents the depolarization of the left and right atria. The P-wave indices could reflect underlying atrial structure, size and electrical activity [16]. The regional delay in atrial depolarization might produce different PWD, as surface P-waves at various locations might be affected to varying degrees by regional variations in atrial activation time. This regional hypothesis explaining variations in PWD intervals was termed Pd [17]. Previous studies have shown that Pd has been recognized as a specific indicator of atrial abnormalities at the electrophysiological and anatomical levels [18]. Marks *et al*. [19] showed that Pd >40 ms was found to be the only independent correlate predicting the risk of AF by following 178 patients with cryptogenic stroke using implantable loop recorders for one year. A recent study of patients with early-onset hypertension identified Pd as a potential electrophysiologic parameter for predicting NAF in this population [20]. In agreement with prior findings, this study showed that increased Pd in sinus rhythm was associated with the risk of NAF within one year.

Pd reflects the prolongation of intra-atrial and interatrial conduction time, as well as nonuniform propagation of sinus impulses within the atria [21]. An increase in Pd signifies a shortened atrial effective refractory period and heightened spatial heterogeneity, accompanied by a loss of the normal heart rate dependence and adaptability. Slowed heterogeneous conduction and increased dispersion of refractoriness within atrial tissue render it highly susceptible to reentry, which can progress to AF in severe cases. A study [22] has reported that atrial dilation can cause atrial myofibrils to become stretched and deformed, leading to their uneven distribution. This subsequently results in non-uniform conduction of atrial depolarization, creating conditions conducive to the unidirectional block and areas of slow conduction required for AF initiation. Therefore, this study incorporated the relevant LAD as a covariate in the regression model. After comprehensive adjustment for confounding factors, the results demonstrated that Pd remained significantly associated with NAF and could serve as an independent predictor. The association between Pd and NAF may help clinicians identify high-risk populations and provides reference for clinical prevention and treatment of AF.

## 5. Limitations

This study has several limitations. Firstly, it was a retrospective study derived from a single center. However, both the cases and controls were selected from a well-defined cohort, which minimized the potential for selection bias. Secondly, although potential confounding factors were adjusted for during the analysis, residual confounding factors may still exist, as is the case with any observational study. Finally, this study cannot yet be compared with other scoring schemes in terms of predictive efficacy for NAF. As mentioned in the discussion section, comparisons between different studies are inherently limited due to variations in study design and study populations. Therefore, future retrospective cohort or prospective studies may incorporate Pd into models such as CHARGE-AF to enhance diagnostic performance.

## 6. Conclusions

In summary, this study demonstrated that increased ECG Pd in sinus rhythm correlated with NAF within one year. After fully adjusting for confounding factors, Pd remained significantly associated with NAF and served as an independent predictor of this risk. This finding may assist clinicians in implementing more frequent follow-up examinations or long-term monitoring for high-risk patients, thereby facilitating early detection of AF.

## Availability of Data and Materials

The datasets used and analyzed during the current study are available from the corresponding author on reasonable request.
